# Life in The Context of Order and Complexity

**DOI:** 10.3390/life10010005

**Published:** 2020-01-18

**Authors:** Christian Mayer

**Affiliations:** Institute of Physical Chemistry, CENIDE, University of Duisburg-Essen, 45141 Essen, Germany; christian.mayer@uni-due.de; Tel.: +49-0201-183-2570

**Keywords:** order, complexity, origin of life, evolution, molecular evolution, prebiotic chemistry, biosignature

## Abstract

It is generally accepted that life requires structural complexity. However, a chaotic mixture of organic compounds like the one formed by extensive reaction sequences over time may be extremely complex, but could just represent a static asphalt-like dead end situation. Likewise, it is accepted that life requires a certain degree of structural order. However, even extremely ordered structures like mineral crystals show no tendency to be alive. So neither complexity nor order alone can characterize a living organism. In order to come close to life, and in order for life to develop to higher organisms, both conditions have to be fulfilled and advanced simultaneously. Only a combination of the two requirements, complexity and structural order, can mark the difference between living and dead matter. It is essential for the development of prebiotic chemistry into life and characterizes the course and the result of Darwinian evolution. For this reason, it is worthwhile to define complexity and order as an essential pair of characteristics of life and to use them as fundamental parameters to evaluate early steps in prebiotic development. A combination of high order and high complexity also represents a universal type of biosignature which could be used to identify unknown forms of life or remnants thereof.

## 1. Estimating System Complexity

Among the many approaches to determine system complexity as a parameter, the idea originally developed by Andrey Nikolaevich Kolmogorov seems to be most appropriate to characterize life [1,2,3,4]. It is based on the assumption that, for every structure, there is a minimal size of an algorithm (or computer program) which fully describes its entity and all of its details. The size of this computer algorithm in a universal description language in bytes may serve as a measure for the degree of complexity of the system. Even though it is hard or even impossible to determine the minimal size of the algorithm exactly (a problem known as Chaitin’s incompleteness theorem [5]), this number still can be approached and estimated for a given system.

**Figure 1:**
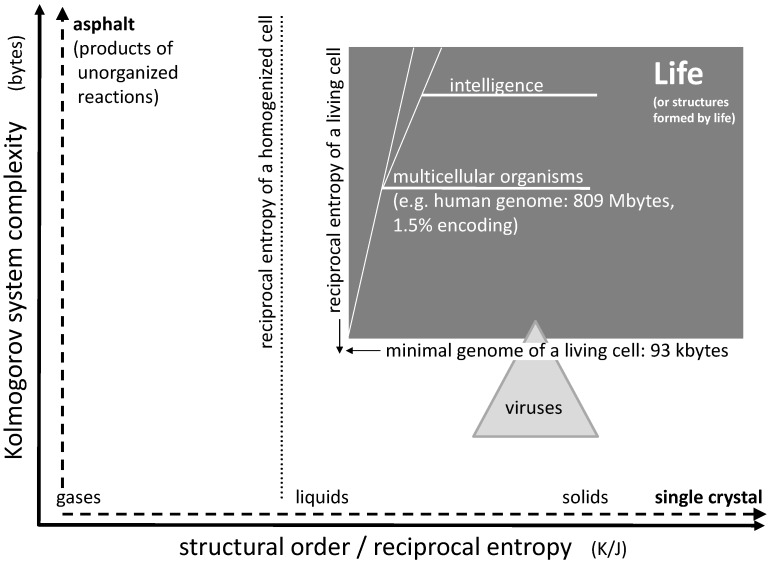
Diagram showing life and other systems in the context of complexity and order. The lower limits of the field of life are defined by the information content of the smallest genome of microorganisms and by the minimum of the reciprocal entropy of a living cell. Every known living organism falls into this category. On the other hand, every single system found in this category is either life itself or a structure formed by life.

As an example, let us consider a sodium chloride crystal. Even if this crystal is of microscopic size, it may still take Tbytes to describe the position of every single ion in space. However, using a suitable computer algorithm, one may derive every single ion position by simply programming a three-dimensional repetition of the cubic elementary cell limited by the outer dimensions of the crystals. Such a computer program would include loops which are repeated over and over, but basically contain the same information as the one for a single elementary cell. Hence, it may actually be reduced to a few bytes. This means that a single crystal of e.g., sodium chloride is actually very low in structural complexity (Figure 1, horizontal axis). In case of a fluid phase state such as liquids and gases, the given complexity approach does not focus on the temporary position and orientation of individual molecules. Instead, it accounts for time averaging by transversal and rotational diffusion, such that these states are of very low complexity as well (the time scale of averaging being adapted to biochemical rate constants).

Chaotic mixtures on the other hand may be very complex. Considering a mix of organic educts undergoing numerous reaction sequences, including oligo- and (co-)polymerization, one may easily end up with millions of reaction products forming an asphalt-like mixture. A corresponding computer program fully describing this mixture would have to account for the structure of each single constituent together with the corresponding concentration. In case of oligomers and polymers, it would have to include every single chain length, every isomer, and every local chemical variation. Therefore, it could easily be of the size of tens or hundreds of Mbytes (Figure 1, vertical axis).

If we consider a living organism, then its genome may serve as a rough representation of an algorithm suitable for its reproduction. Even though the genome may contain unknown junk (non-coding) sequences and even though the reproduction of the organism requires additional structures with additional complexity, the size of the essential genome in bytes gives an idea about the organism’s complexity. An example for a natural organism with an extremely small genome is *Nanoarchaeum equitans*, a hyperthermophilic and possibly parasitic archaeon [6]. Its DNA consists of just 490,885 base pairs, corresponding to 981,770 bits or, with one byte equal to eight bit, roughly 123 kbytes. This may be close to the lower limit of life’s complexity. Organisms with smaller genomes may exist, but they are usually depending on biomolecules in their environment or on other organisms, as in case of viruses. Of course, multicellular organisms are of significantly higher complexity. A human genome consists of approximately three billion base pairs which comprise about 809 Mbyte of stored information. As little as 1.5% of this information may be transformed into protein structures, while the vast majority is reserved for regulation of gene expression, for chromosome architecture or may not serve any purpose at all [7]. So the actual value for complexity of the human genome may be somewhere between 12 and 800 Mbytes, which of course is a very rough estimation. However, this is at least a hundred times larger than the value for *Nanoarchaeum equitans* mentioned above.

## 2. Estimating Structural Order

The term “order” refers to the state of a system from a largely human perspective and is, by itself, largely undefined and also depends on individual judgement. For the purpose of this consideration, we rather want to focus on a corresponding thermodynamic parameter which is the entropy of the system. The entropy (S) is given in units of J/K and may be defined thermodynamically as well as statistically. It unambiguously describes the state of a system regarding its disorder, even though it may be difficult to be determined in practice and even though the outcome may occasionally deviate from the human interpretation of disorder. In the following and for simplicity, we want to assign the parameter “structural order” to the reciprocal system entropy (S^−1^). Doing so, the structural order of a system within given local boundaries can be estimated in units of K/J.

Considering single-component systems, the structural order (or reciprocal entropy) largely depends on the phase state (Figure 1, horizontal axis). It distinctly increases from the gaseous state via the liquid state to the solid state. The ultimate structural order is represented by the crystalline state: Virtually nothing is more ordered than a single crystal of e.g., sodium chloride at zero temperature. Likewise, the other end of the scale could be represented by a gas at high temperature. On the scale between these two extremes, the structural order (i.e., the reciprocal entropy) changes gradually with temperature and abruptly at the points of phase transitions.

The reciprocal entropy of a living cell is principally accessible by calculation. It is larger than the one of a liquid solution of all cell constituents but smaller than the one of an amorphous gel-like solid formed under cross-linking of all polymer ingredients (Figure 1). Its entropy differs from the one of a homogenized counterpart of the cell by all mixing entropies which would be obtained after every single membrane or other separating boundary has been disassembled and all intracellular concentration gradients have vanished. The corresponding drop of structural order basically separates a living cell from a dead one: the initial process connected to the beginning death of a cell is the disappearance of a trans-membrane concentration gradient connected to the lysis of any internal or external cell membrane. Rather than calculating the actual absolute value of the reciprocal entropy, the corresponding value for the homogenized cell (or tissue) may serve as a reference point for the estimation of the structural order of a living organism (Figure 1).

It is important to note that the term entropy used for this definition is by no means related to the *entropy of the information source* which can be derived from the Kolmogorov complexity [4]. In the context of this discussion, complexity and order (as reciprocal entropy) are completely independent parameters.

## 3. Order and Complexity: Boundaries of Life

With the definition of system complexity and structural order given above, one may try to roughly localize the boundaries for living organisms (Figure 1). Regarding structural order, the typical reciprocal entropy of a living cell represents the marker for life. Even a small loss of this order, e.g., connected to a disrupted membrane, can mean the actual death of the cell. Hence, the borderline between life and death can consist in the mixing entropy of two media originally separated by a membrane. If the entropy of a homogenized cell is used as a reference, the sum of all mixing entropies connected to the homogenization of the cell would define the boundary for the structural order of life. It is the first line that is rapidly crossed during the acute death of an organism. Some uncertainty of the position of this borderline may be induced by dehydration or swelling of a cell (e.g., caused by osmosis), but this variation is limited. An upper limit for structural order of life does not exist.

Regarding complexity of biological life, the lower limit is defined by the smallest genomes of living microorganisms as already mentioned above. This boundary is somewhat diffused as some of these organisms tend to rely on biomolecules (e.g., lipids) in their environment and may not be regarded as independent life. Generally, the minimum number of genes sufficient for cellular life has been estimated to be 300 which, assuming 1.25 kbp per gene, corresponds to 375 kbp or approximately 93 kbytes [8,9,10]. 

An average virus genome is about 40 kbytes in size, hence the complexity of a typical virus is actually well below this limit. However, in extreme cases, virus DNA may also be as large as 560 kbp corresponding to 140 kbytes [8], a value significantly above the lower limit of cellular life. The so called Pandoraviruses have even been discussed to represent a fourth domain of life next to Bacteria, Archaea, and Eukaryotes. Therefore, there is a small but remarkable overlap between the fields of viruses and cellular life. In terms of structural order, viruses generally exceed the values of cellular life as they often exhibit more regular geometries than living cells (Figure 1).

Multicellular life tends to be more complex as well as slightly more ordered than single cells. The cellular architecture requires additional information as well as a certain additional quantum of reciprocal entropy, the latter being represented by the mixing entropy of cells connected to the disintegration of an organized tissue (without destroying the cells). An additional quantity of complexity develops in higher organisms when they develop memory and intelligence during their lifetime. An upper limit for complexity of life does not exist.

## 4. Functional Processes of Life

Clearly, life cannot proliferate without functional processes. These processes mainly consist of metabolic reactions that occur in the state of non-equilibrium. All these steps proceed irreversibly and under reduction of the free enthalpy of the system (ΔG < 0), following the second law of thermodynamics. If we consider a cell that is completely isolated from its environment, i.e., with no exchange of material or energy, such a process necessarily leads to its death. Under these circumstances, the entropy of the cell would increase continuously, otherwise the process would lack its driving force. Increasing entropy of the cell however means decreasing structural order, so in the context of Figure 1, the system would follow a horizontal path to the left and eventually leave the area of life.

In order to survive any process, the cell must “feed on negative entropy,” as stated by Erwin Schrödinger in the original edition of his book “What is Life” [11,12]. The most efficient way to do so is to dissipate energy [12]. By emitting heat to the environment in an exothermic process (ΔH < 0), the system creates a positive entropy change in the surrounding medium and hereby a negative contribution to the system’s free enthalpy (with ΔG = ΔH − T × ΔS at constant temperature). This given, the cell can keep or even decrease its own entropy S (“eat negative entropy”) and avoid the loss of order. Actually, this mechanism is one of the main reasons why a living cell as we know it needs energy to survive (the others may concern mobility, growth or variation of the cell’s surroundings). A good example for this is the activation of monomers by the formation of pyrophosphates. The chemical energy of the pyrophosphate (e.g., ATP) and the subsequent release of heat drives condensation processes which otherwise would not be favorable since they may be connected to a portion of negative entropy. This given, an important contribution of metabolic processes is the preservation of the structural order of the cell, avoiding a horizontal shift to the left in the order/complexity diagram. In addition, the multiplication of single cells or whole organisms would not be possible without cell metabolism. However, as the given model applies to living entities, reproduction itself is not under consideration.

Regarding the vertical axis in the diagram, the multitude of processes clearly contributes to the complexity of a living cell. Obviously, the large number of non-equilibrium processes presents a significant part of the evident complexity of life. However, all these processes are basically coded in the cell’s genome which is responsible for the entity of all enzymes. All in all, it is this collection of enzymes which fully controls the network of the cell’s metabolism, down to every single non-equilibrium process. In other words: in the sense of the Kolmogorov approach, the genome represents the computer program for the full metabolism. For this reason, the corresponding contribution to complexity is reflected by the one of the genome and therefore is part of this model.

Finally, we have to consider life without a functional process. Bacterial spores do not exhibit any signs for metabolism, but they can principally survive for millions of years [13]. Alternatively, metabolic processes in living cells can virtually be stopped by storage in liquid nitrogen: structurally intact living cells and tissues are well preserved by freezing under suitable conditions [14]. These forms of dormant life are accounted for in the given model, since they keep their full system complexity and remain in their original structural order (which may even be increased by removal or freezing of the aqueous phase).

## 5. Order and Complexity as a Biosignature

The area of life indicated in Figure 1 may be regarded as a very fundamental biosignature: every life form known on earth would be located within the given boundaries. On the other hand, every single structure on earth fitting into the given boundaries is either life itself or a structure formed by life. The first category is given for uni- or multicellular organisms, the second for complex shells of diatoms, the nest structures formed by insect colonies, a book, a computer program, a painting, a musical composition, social structures or artificial intelligence. So every structure beyond a given complexity and a given order can be attributed to life, either directly or indirectly. 

At this point, it is important that the high structural order and high complexity form a functional unit (see Section 4). A system composed of a mineral crystal next to a chunk of asphalt would not fulfill this condition, as the two materials form two strictly separate domains with no functional connection in between.

## 6. Evolution: A Powerful Mechanism Driving Order and Complexity

Several natural processes can lead to a spontaneous increase of structural order in a given system (while an external environment is decreasing its structural order to fulfill the second law of thermodynamics). As an example, a highly ordered salt crystal can form spontaneously from an oversaturated solution (while water molecules are evaporated). Following a horizontal line in the order/complexity diagram is easy.

Several natural processes can lead to a spontaneous increase of a system’s complexity. A mixture of organic compounds when heated and bombarded with UV light may undergo numerous reactions including polymerization processes, leading to a very large number of products. The final result may resemble asphalt and may consist of hundreds of thousands of compounds [15]. Following a vertical line in the order/complexity diagram is quite easy as well.

**Figure 2:**
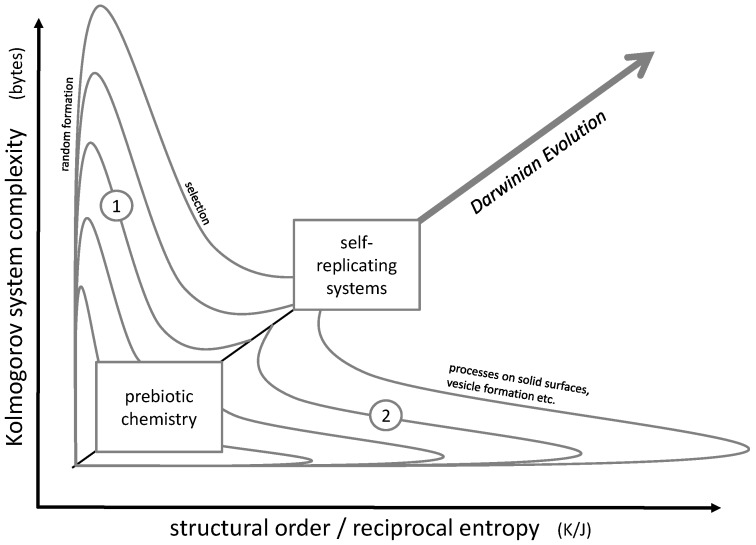
Diagram representing the course of prebiotic evolution in the context of order and complexity. The general target of an evolution process is the simultaneous increase of both parameters. In detail, possible pathways include an initial increase of random variability followed by selection (lines 1) or the transition through a highly ordered structure (like micelles, surfaces or crystals) followed by an increase of complexity (lines 2).

The actual challenge consists in the simultaneous increase of order and complexity, in other words, advancing along a diagonal line in the order/complexity diagram (Figure 2). The most powerful natural approach to accomplish this task may be Darwinian evolution. It obviously led from microorganisms to the most complex forms of life, their complexity being accompanied by a high degree of cellular order in multiple tissue structures. Even though it does not always follow a straight line along the diagonal of the order/complexity scheme, it still shows a clear tendency in this direction.

In principle, Darwinian evolution approaches the diagonal pathway by initially producing complexity (e.g., random genetic variation by mutation), then increasing order and simultaneously losing complexity by a selection process. This pathway could be described by lines 1 in Figure 2.

## 7. Consequences for Prebiotic Chemistry

This principle should also hold for all developments prior to the formation of the first living cell, such as for prebiotic chemistry. Processes equal or similar to Darwinian evolution may also occur on a molecular scale, but this necessarily requires complex chemical reaction networks [16,17] or molecules with the capability to undergo self-reproduction. The most prominent representative for a self-replicating system may be ribonucleic acid (RNA), which is the essential part of the well-known RNA world hypothesis [18,19,20].

However, this stage of chemical evolution [21] could require a well-developed structural molecular environment, including compartimentation [22,23] and energy metabolism. An efficient non-Darwinian pathway for the development of a suitable starting point for the RNA world could have been based on random formation and selection. Random formation of oligomers, e.g., during wet-dry cycling, would initially increase the system’s complexity [24,25]. In the subsequent step of selection, the complexity would decrease while structural order would build up. In Figure 2, such a development would be represented by lines 1. 

Alternatively, complex and ordered structures may be generated by an initial formation of highly ordered structures (such as crystals), which then develop into complexity (lines 2 in Figure 2). Examples for this principle would be the initial formation of (relatively ordered) micelles which then transform into (more complex but less ordered) vesicles [26,27], a complex evolution of self-organized amphiphiles (known as the GARD model) [28] or early metabolic steps which could have started on mineral surfaces and then could have been transferred into vesicles and cells [29,30].

Both pathways 1 and 2 may also have been followed iteratively in subsequent steps. A corresponding model was recently proposed by C.W. Carter and P.R. Wills: it involves a close interaction between peptides and RNA strands undergoing a co-evolution process [31,32,33,34,35,36]. Here, an initial selection process (represented by pathway 1) is followed by a spontaneous formation of a peptide-RNA-complex passing through a maximum of order. In the following steps, order decreases again while complexity rises (represented by pathway 2). A similar example for an iterative approach involving pathways 1 and 2 may be seen in a co-evolution process of peptides and vesicles which was observed experimentally [37]. Here, a peptide selection process (represented by pathway 1) is followed by the formation of mixed micelles, which due to the mixing process lose their original order while gaining complexity (pathway 2).

In any case, the development of early prebiotic chemistry into self-replicating systems necessarily had to follow the diagonal line in Figure 2. All in all, this diagonal could mark the general direction of all steps which finally lead to the formation of the first living cell. At a certain point, the development crosses the limits of biological life discussed in Section 3 and—according to the general idea of this article—reaches the state of a living organism.
